# Give It a Name

**Published:** 1892-01

**Authors:** 


### GIVE IT A NAME.

OFFICE OF J. H. CHAMBERS & CO., PUB. ANNALS SURGERY,
ST. LOUIS, MO., DEC. 1891.

*Editor Texas Med. Journal, Austin, Texas:*

DEAR DOCTOR—We have mailed you the "Annals of Surgery" during the present year, in lieu of which you were to mention this journal editorially three times during the year, at least this is our understanding. We would like to have you advise us if such editorial mention has been made; if so, in what numbers of your journal? The expense of producing a special journal, such as the "Annals," as you are probably aware, amounts to quite a large item during the year, and as indicated in our previous letters, your journal is not essential to the editor of the "Annals of Surgery." If you have not already made editorial mention of the "Annals" recently, kindly do so in your next issue, and advise us if you desire that we should mail the "Annals" to you during the coming year on the terms herein indicated.

With best wishes, we remain, Yours Truly,

J. H. CHAMBERS & CO.

The above, we presume, is a circular letter. It will be remembered that last year and the year before, Mr. Chambers issued a similar circular to the American medical press, only, in 1889 he said "whereas our editor has no use for such journals" (the American medical exchanges) the *Annals* would be sent only to those who gave "editorial notices three times a year of the *Annals*."

The letter of 1889 was published in the *Buffalo Medical Journal* under the head "amusing effrontey." DANIEL'S TEXAS MEDICAL JOURNAL also gave it a "benefit," the effect of which was to bring from Mr. Chambers a severe letter, in which he promised, however, that in consideration of our free notice he would make an exception of us, and place our "esteemed journal" on the free list. We now get the above. Mr. Chambers' memory is not good. This is the only reply we make: we "scratch" the *Annals* and the *Annals*, alas! will have to scratch "we." (We weep.)

As Mr. Bailey said to Capt. Cuttles—"give it a name;" somebody give a name to the extraordinary course pursued by Mr. Chambers. It is not effrontery; it is not "cheek;" it is not ignonorance. Mr. C. is so in love with his *Annals* that like a doting parent, he sees excellencies in it not visible to less partial eyes. It is only a monthly, a 96 page, very modest looking pamphlet. There are scores of better, sent freely in exchange with even the least pretentious confreres. Why should the *Annals* arrogate to itself such superiority, and not only demand to be "puffed" in order to get it, but the publisher must slap all others in the face and refuse them, as "useless" to his editor. Had the *Annals* been issued on its merits alone, if it possess any, exchanges would have given it the notice it deserves. Its articles, if meritorious, would be copied and credited. The course Mr. C. has seen fit to pursue cannot have been dictated by that spirit of courtesy which is supposed, at least, to characterize the medical press.

